# Expression and characterization of recombinant VP19c protein and N-terminal from duck enteritis virus

**DOI:** 10.1186/1743-422X-8-82

**Published:** 2011-02-24

**Authors:** Jun Xiang, Shunchuan Zhang, Anchun Cheng, Mingshu Wang, Hua Chang, Chanjuan Shen, Dekang Zhu, Renyong Jia, Qihui Luo, Zhengli Chen, Xiaoyue Chen

**Affiliations:** 1 Avian Diseases Research Center, College of Veterinary Medicine of Sichuan Agricultural University,Ya'an, Sichuan, PR China; 2 Key Laboratory of Animal Diseases and Human Health of Sichuan Province, Ya'an, Sichuan, PR China; 3 Epizootic Diseases Institute of Sichuan Agricultural University, Ya'an, Sichuan, PR China

## Abstract

**Background:**

Previous studies have indicated that the VP19c protein and its homology play similar roles in capsid assembly of all *Alphaherpesvirus *subfamily. However, there is no report on the VP19c protein of duck enteritis virus (DEV). In this study, we expressed the DEV VP19c protein and presented its antigenic properties. Moreover, we developed polyclonal antibody against the VP19c protein and characterized it.

**Methods:**

A recombinant VP19c (rVP19c) and N-terminal were expressed in *Escherichia coli *(E.coli) and purified by Ni2+-affinity chromatography. The antigenic properties of the recombinant protein were determined by Western blot and indirect enzyme-linked immunosorbent assay (ELISA). Furthermore, the polyclonal antibodies against the purified recombinant proteins were produced and the titer of polyclonal antibody was determined by ELISA analysis. Finally, the antibody was used to recognize the VP19c in the cells infected with DEV in the immunofluorescence assay.

**Results:**

The N-terminally His-tagged rVP19c and rVP19c(N) were produced as inclusion bodies in E. coli strain BL21 (DE3) with molecular weight of about 66 and 46 kDa. Then the proteins were purified to reach the level of homogeneity. Western blot and ELISA analysis that the rVP19c seems to be structurally and antigenically very similar to native VP19c and the N-terminus of VP19c may contain most antigenic linear-epitopes. Furthermore, ELISA analysis demonstrated that the titer of polyclonal antibody was approximately 1:12800, and in the immunofluorescence assay, the antibody was able to recognize the VP19c in the cells infected with DEV.

**Conclusions:**

To our knowledge, this was the first report on basic properties of DEV VP19c protein. In the present study, we obtained a high-level expression of the recombinant VP19c protein as well as high titers of rabbit polyclonal antibody against to VP19c protein. The anti-rVP19c serum was able to detect the VP19c protein in DEV infected cells and the VP19c protein targeted to the nucleus as distinct punctate speckles. This specific polyclonal antibody provides a good tool for further studying structural and functional characterization of DEV VP19c.

## Background

Duck viral enteritis (DVE) is an acute, contagious, and lethal disease of waterfowl of the family Anatidae worldwide [1]. The causative agent, duck enteritis virus (DEV), is a member of the family *Herpesviridae*, in which herpes simplex virus type 1 (HSV-1) is studied most completely. While research on molecular epidemiology of DEV has advanced over the years [2], relatively little is known concerning the structural, functional and immunogenic role of the structural proteins. The DEV virion is enveloped and the genome consists of double-stranded DNA segments packaged in an icosahedral capsid of several structural proteins [3]. The genetic information of viruses is enclosed in a capsid shell, a protein coat whose function is to protect the nucleic acid and to aid in the infectious process. In the HSV-1, capsid is an icosahedral shell, three of whose primary structural components are a major capsid protein (VP5; coded by the UL19 gene) and two minor capsid proteins, VP19c (UL38 gene) and VP23 (UL18 gene). VP19c and VP23 make up the triplex, which plays an essential role in capsid assembly and architecture [4]. Cell localization studies have also demonstrated the requirement of VP19c for the nuclear localization of VP23 [5]. Interestingly, the HSV-1 UL38 is regulated with late kinetics [6], whereas the bovine herpesvirus type 1 (BHV-1) and pseudorabies virus (PRV) UL38 transcript belong to the early kinetic class [7,8]. Most of the information of DEV UL38 gene currently is from bioinformatic approaches. Lacking an antibody against DEV VP19c, studies on biofunctions related to it are limited.

Computational predictions of the VP19c amino acid sequence revealed that epitopes were more abundant on the N-terminal half of the VP19c protein than the C-terminal half of it [9]. Hence, in the present study, partial and full-length coding open reading frame (ORF) of UL38 gene were cloned, for the first time, into pET-32a(+) expression vector to obtain abundant recombinant proteins in E. coli. Moreover, their antigenic properties were characterized by western blot analysis and ELISA. Subsequently, two polyclonal antibodies were raised against the purified recombinant proteins in rabbits, and the titer and specificity of the polyclonal antibodies were characterized further by ELISA and immunofluorescent assays.

## Results

### Expression and purification of recombinant DEV VP19c and VP19c(N)

**Figure 1:**
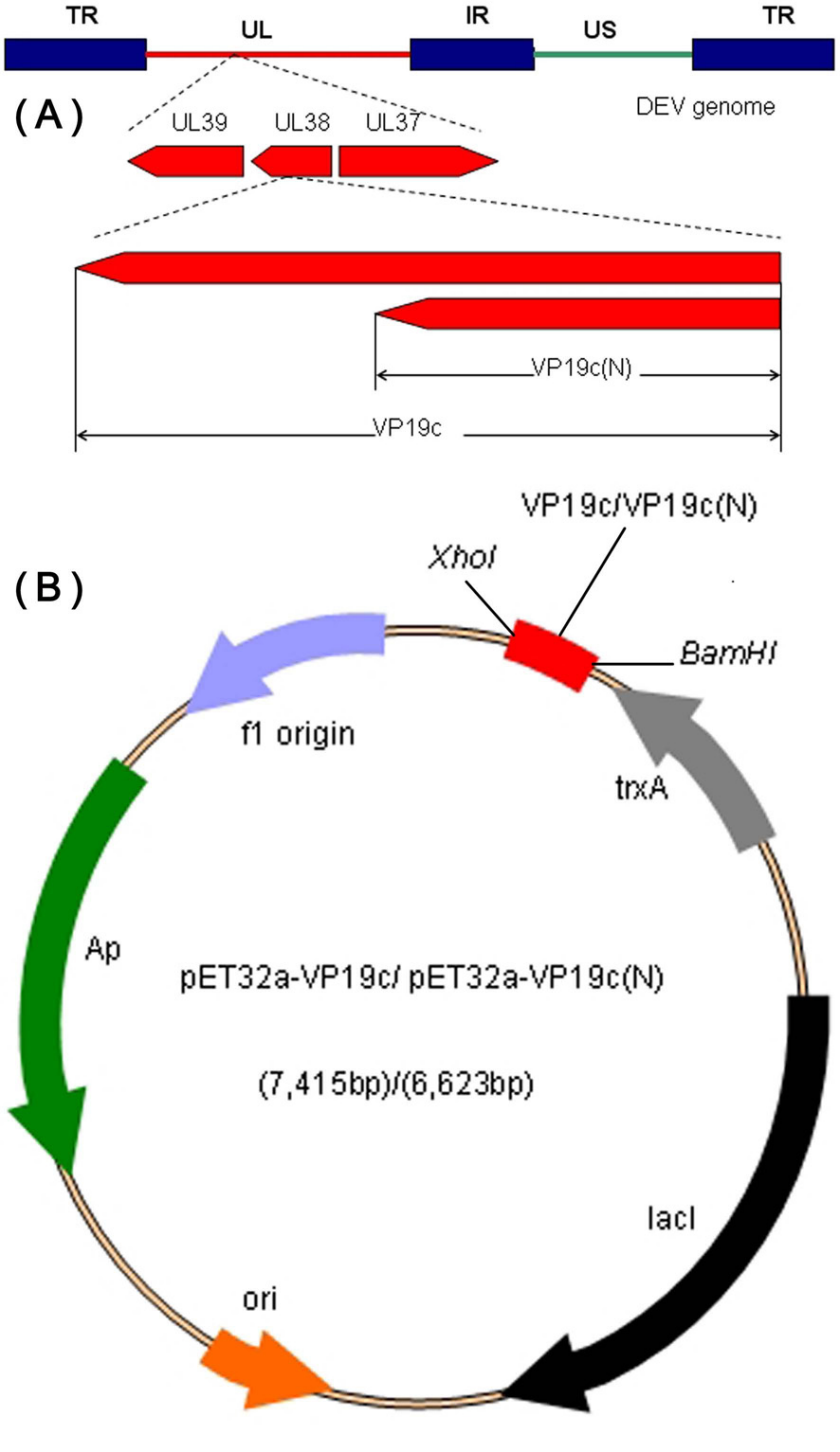
**Strategy of vector construction**. (A) Scheme of the arrangement of UL38 gene in DEV genome and the PCR amplification strategy. (B) Map of plasmid pET32a-VP19c and pET32a-VP19c(N).

**Figure 2:**
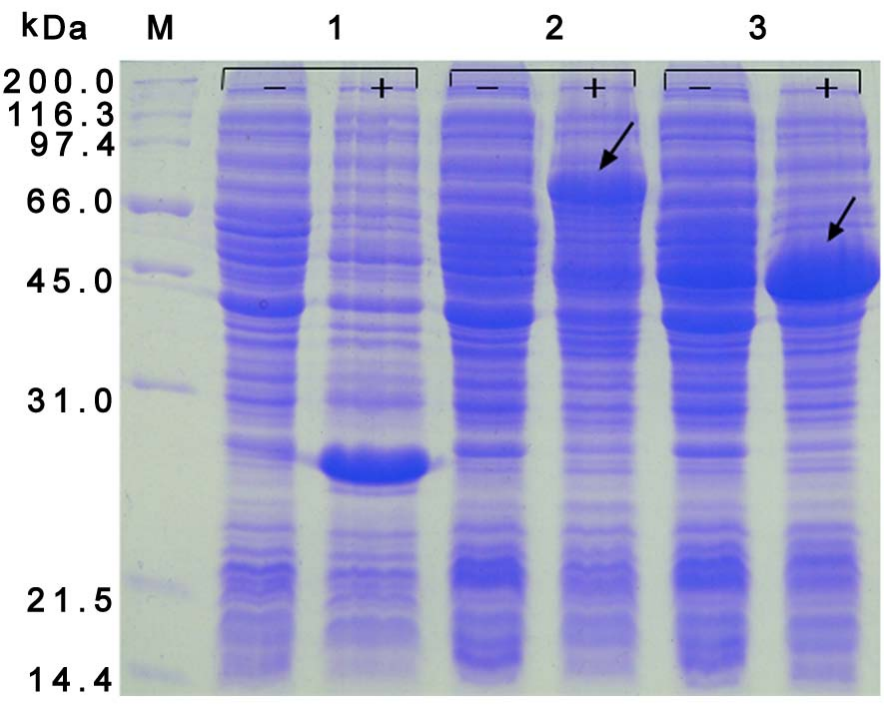
**SDS-PAGE analysis of recombinant proteins**. M, wide molecular weight protein marker; 1, control E. coli cell transformed without the insert; 2, the whole bacterium of pET32a-VP19c; 3, the whole bacterium of pET32a-VP19c(N). All of strains were grown in the presence (+) or in the absence (-) of IPTG.

**Figure 3:**
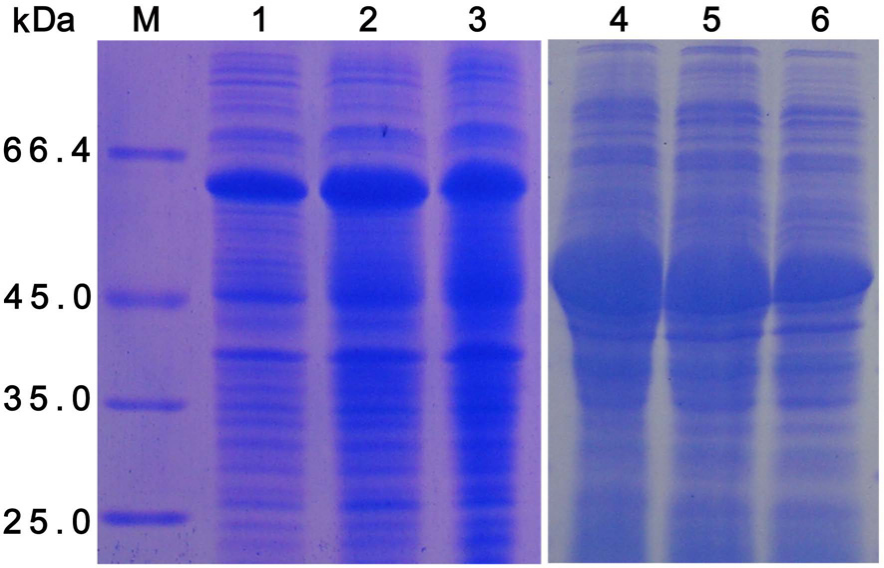
**Temperature optimization analysis by SDS-PAGE**. M, low molecular protein marker; 1, 2, and 3, the bacterial cultures of pET32a-VP19c were induced in 25°C, 30°C, 37°C, respectively; 4, 5, and 6, the bacterial cultures of pET32a-VP19c(N) were induced in 37°C, 30°C, 25°C, respectively.

**Figure 4:**
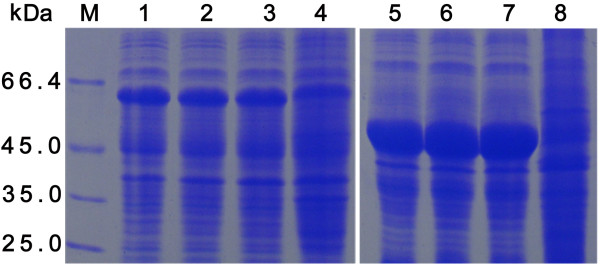
**Time optimization analysis by SDS-PAGE**. M, low molecular protein marker; 1, 2, 3, and 4, the bacterial cultures of pET32a-VP19c were induced for 3 h, 4 h, 5 h, overnight (16 h), respectively; 5, 6, 7, and 8, the bacterial cultures of pET32a-VP19c(N) were induced for 3 h, 4 h, 5 h, overnight (16 h), respectively.

The cloning strategy for constructing the recombinant plasmids is shown in Figure 1. The N-terminally His-tagged rVP19c and rVP19c(N) were produced in E. coli strain BL21 (DE3) with molecular weight of about 66 and 46 kDa (Figure 2). The optimal temperatures for rVP19c and rVP19c(N) expression were 30°C and 37°C (Figure 3) respectively, and optimal induction times for them were both about 4h (Figure 4), while concentrations of IPTG (data not shown) had a little influence on their expression. With the analysis of software BandScan 5.0, the induced rVP19c and rVP19c(N) reached approximately 40% and 70% in the total bacteria protein of each 1 ml culture, respectively.

**Figure 5:**
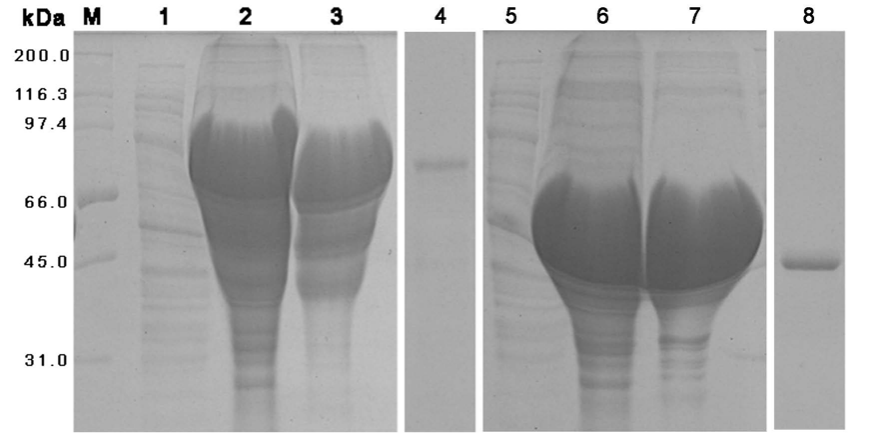
**Analysis of solubility and purification of recombinant protein by SDS-PAGE**. M, wide molecular weight protein marker; 1, the supernatant of induced pET32a-VP19c after sonication; 2, the pellet of induced pET32a-VP19c after sonication; 3, the inclusion bodies of rVP19c by rough extraction; 4, the purified rVP19c by Ni-NTA affinity column; 5, the supernatant of induced pET32a-VP19c(N) after sonication; 6, the pellet of induced pET32a-VP19c(N) after sonication; 7, the inclusion bodies of rVP19c(N) by rough extraction; 8, the purified rVP19c(N) by Ni-NTA affinity column.

The rVP19c and rVP19c(N) (Figure 5) were successfully expressed as inclusion bodies in E. coli and isolated roughly. The final yields of denatured soluble inclusion bodies were estimated to be approximately 100 mg/L (rVP19c) and 150 mg/L (rVP19c(N)) of initial bacterial culture. Then the proteins were purified by IMAC under denaturing conditions to reach the level of homogeneity (Figure 5).

### Antigenicities analysis of rVP19c and rVP19c(N)

**Figure 6:**
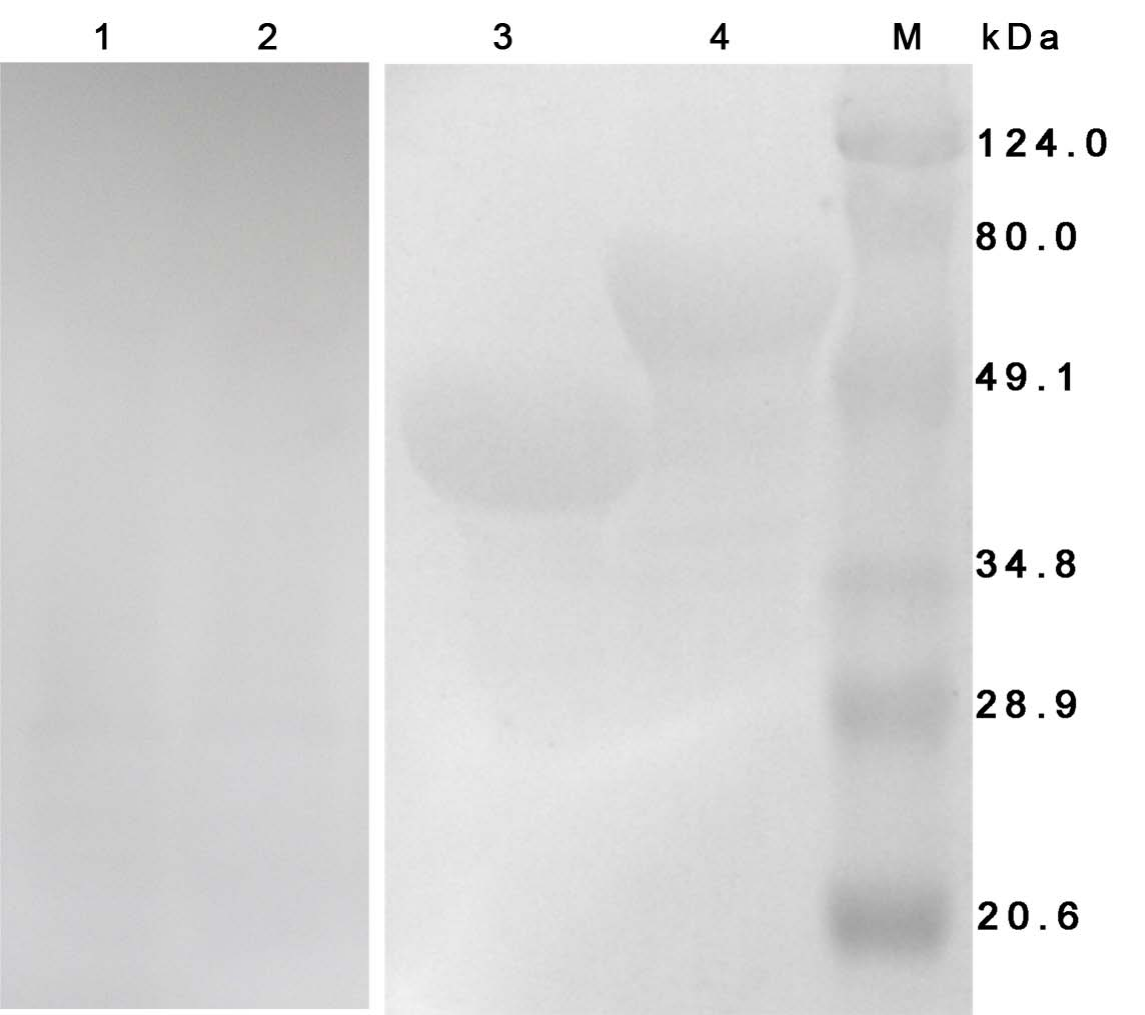
**Western blot analysis of recombinant proteins with rabbit polyclonal antibody to DEV**. 1, the rVP19c(N) protein incubated with rabbit pre-serum; 2, the rVP19c protein incubated with rabbit pre-serum; 3, the rVP19c(N) protein incubated with polyclonal anti-DEV antibodies; 4, the rVP19c protein incubated with polyclonal anti-DEV antibodies; M, prestained protein ladder.

**Figure 7:**
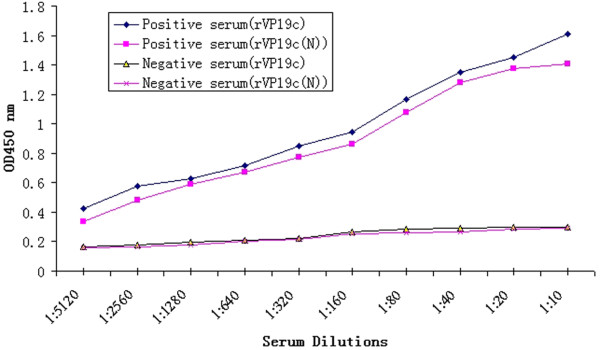
**Antigenic properties analysis**. ELISA analysis of recombinant proteins with duck anti-DEV positive serum. Duck negative serum was used to be a control.

As shown in Figure 6, polyclonal antibody from rabbit immunized against purified DEV reacted with rVP19c and rVP19c(N). The results showed a similar immune-detection pattern as the native VP19c protein presented in the virus and the VP19c(N) was involved in antigenic recognition of lineal-epitopes recognized by PAb under denaturing conditions. In ELISA assay, the positive serum was able to recognize the rVP19c(N) protein at a similar level as rVP19c protein(Figure 7), which indicated that the linear epitopes within VP19c(N) recognized by positive serum were the same in the VP19c protein.

### Characterization of the polyclonal antibodies against rVP19c and rVP19c(N) proteins

The antibodies against rVP19c and rVP19c(N) proteins at different dilutions were reacted with fusion proteins, pre-immunized rabbit serum served as the negative control. The two antibodies titer were found to be approximately 1:12 800. At the same time, negative control did not result in a detectable signal (data not shown).

**Figure 8:**
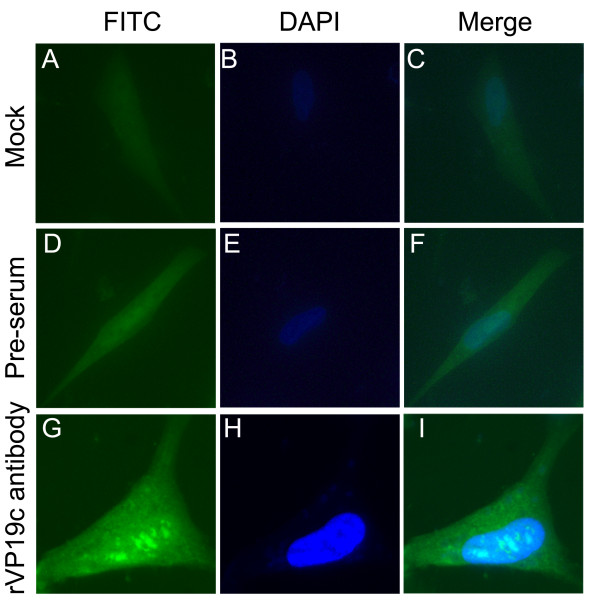
**Indirect immunofluorescent assay of the VP19c protein in DEV infected DEF cells**. Mock infected DEF cells probed with the VP19c antibody (A). DEV infected DEF cells probed with the preimmune sera (D) or VP19c antibody (G). Cells were labeled with FITC-conjugated goat anti-rabbit immunoglobulin G and counterstained with DAPI to visualize the nuclei (B, E, H). Then FITC and DAPI were merged (C, F, I). (Images were acquired by using 60 × objective).

As shown in Figure 8G, anti-rVP19c serum was able to detect the VP19c protein in DEV infected cells and the VP19c protein targeted to the nucleus as distinct punctate speckles. In contrast, there was no staining in mock infected cells (Figure 8A) or DEV infected cells detected with the preimmune serum (Figure 8D). Similarly, the antibody against rVP19c(N) was able to recognize the VP19c protein in the cells infected with DEV (data not shown).

## Discussion

Currently, morphological features [3,10] and complete genome sequence [11] of DEV have been defined, but studies on proteins of DEV virion, especially structural and functional characteristics of viral structural proteins, have been very few. Generally, the structural viral proteins have various functions during the replicative cycle, which might be closely related to their structure [12]. Therefore, the understanding of the function of each structural protein depends on the study at a molecular level.

Computational predictions of the VP19c amino acid sequence suggested that it did not require any post-translational modifications and lacked transmembrane regions and signal peptides, so it is more suitable for prokaryotic expression. And predictions presented the N-terminus of VP19c protein may contain most antigenic linear-epitopes [9].

To obtain high-level expression, several expression conditions, such as induction time, induction temperature, and IPTG concentration were optimized. It could be seen that temperature of induction is critical. After induction conditions optimized, the expected high-level expression result was obtained.

Western blot confirmed that the expressed recombinant proteins were specific to DEV and had good antigenicity. These results strongly suggested that the purified rVP19c protein seemed to be structurally and antigenically very similar to native VP19c protein and the antigenicity of VP19c was also mainly determined by linear antigenic determinant of it. Inclusion bodies (IBs) are denatured proteins and lack conformational epitopes. Thus it can be seen that though the IBs are inactivated form of protein, they are sufficient for animal immunity and harvest of specific sera [13]. Moreover, the rVP19c(N) was deleted C-terminus, but still remained good antigenicity. The results of ELISA assays showed that the antibody in the duck sera raised against the complete VP19c protein were primarily against the N-terminal region of the VP19c protein, which indicated that the N-terminal portion of the VP19c protein may harbor most of the linear epitopes. Furthermore, anti-rVP19c serum was able to detect the VP19c protein in DEV infected cells, which indicated that this specific polyclonal antibody provide a good tool for further studying structural and functional characterization of DEV VP19c.

Currently, little is known about DEV VP19c function or regulation of expression, as well as other features. Homology analysis with other herpesviruses suggested that the DEV VP19c protein could be a crucial capsid protein [14]. Conservation of the protein suggests that it may play a role in the viral life cycle. Because of the localization of VP19c to viral factories, we investigated the possibility that this protein might be incorporated into DEV virions.

## Conclusions

To our knowledge, this was the first report on basic properties of DEV VP19c protein. In the present study, we obtained a high-level expression of the recombinant VP19c protein as well as high titers of rabbit polyclonal antibody against to VP19c protein. The anti-rVP19c serum was able to detect the VP19c protein in DEV infected cells and the VP19c protein targeted to the nucleus as distinct punctate speckles. This specific polyclonal antibody provides a good tool for further studying structural and functional characterization of DEV VP19c.

## Methods

### Construction of plasmid

The viral DNA was extracted from partially purified DEV and used as template for polymerase chain reaction (PCR) [15]. A fragment (1515 bp) containing the complete ORF of DEV UL38 gene (1398 bp) (Genbank accession no. EU071041) was amplified using PCR with the following primers: the upstream primer (P1) 5'-GGATCCACGATGAAAGTACCAAATG-3' containing the BamHI site and the downstream primer (P2)5'-CTCGAGAGCCAGATACGAC AAGAAG-3' containing the XhoI site. The PCR parameters were 10 min at 95°C and 30 cycles of 1 min at 94°C, 1 min at 58.5°C, 1 min at 72°C, and a final extension time of 10 min at 72°C. A fragment (723 bp) containing the partial ORF (711 bp) encoding the N-terminal of VP19c (VP19c(N)) protein was amplified with the following primers: the upstream primer (P1) 5'-GGATCCACGATGAAAGTACCAAATG-3' containing the BamHI siteand the downstream primer (P3) 5' -CTCGAGGTGTACGTGACATCTAACCA TAG-3' containing the XhoI site. The PCR assay and programme were carried out following the protocol described above.

The PCR products and plasmid pET-32a(+) (Qiagen GmbH, Hilden, Germany) were both digested with BamH I and Xho I, and then ligated with T4 DNA ligase to yield the constructs. The constructs were transformed into E.coli, and the selected bacterial transformants were verified by colony PCR, restriction enzyme analysis and sequencing.

### Expression

The positive individual clone was cultured in 5 mL Luria bertani (LB) medium containing 100 ug/mL amp and then induced at 37°C by adding isopropyl-β-D-thiogalactoside (IPTG) at a final concentration of 1 mM for 4 h. For IPTG dose optimization, the bacterial culture was induced with different concentrations of IPTG [0.2, 0.3, 0.4, 0.5, 0.6,0.7,0.8,0.9,1.0 (mM)] and allowed to grow for 4h at 37°C. For temperature optimization, the bacterial culture was induced with IPTG [1.0 (mM)] and allowed to grow for 4 h at three different temperatures (25, 30 and 37°C). For time optimization, the bacterial culture was induced with IPTG [1.0 (mM)] and allowed to grow for 3 h, 4 h, 5 h and overnight (~16 h) at 37°C. Total cell proteins from each optimization experiment were analyzed by sodium dodecyl sulfate-polyacrylamide gel electrophoresis (SDS-PAGE). Then, small-scale expression was done by optimized conditions as described above to prepare for purification [16]. The protein amount was determined with reference to standard bovine serum albumin (BSA) in the Bradford assay [17].

### Purification

500 ml induced bacterial culture was harvested after 4 h, centrifuged at 6000 × g for 10 min and the cell pellet was suspended in 20 mM Tris buffer (pH = 8.0). The cells were later lysed by using lysozyme (0.1 mg/mL) at 4°C for 1 h and sonicated on ice for 5 min at an amplitude of 30% with a 30s pulse frequency. The lysate was centrifuged at 10,000 × g for 20 min at 4°C and the supernatant was collected as soluble fraction. The resulting pellet was washed twice with 10 mL 2M urea containing 50 mM Tris buffer (pH = 8.0), 1 mM EDTA, 150 mM NaCl and 0.1% Triton X-100.

The suspension was centrifuged at 10,000 × g for 20 min at 4°C and then the resulting subsidence was resuspended in regeneration buffer containing 6 M urea, 0.5 M NaCl, 20 mM Tris-HCl (pH 7.9) and incubated at room temperature for 30 min. The incubated mixtures were then centrifuged at 10,000 × g for 20 min, and the supernatant was submitted to further purification. The supernatant was then poured on to a purification column and allowed to bind for 1 h with gentle shaking. The recombinant His-tagged proteins were purified from the supernatant obtained above by immobilized metal affinity chromatography (IMAC) on Ni-NTA affinity resin (Bio-Rad) following the conventional protocol [18]. Finally, the proteins were collected and analyzed by SDS-PAGE to assess the level of homogeneity.

### Western blot

The recombinant proteins were electrophoresed with SDS-PAGE using 12% polyacrylamide gel and then electroblotted onto polyvinylidene fluoride (PVDF) membrane. The PVDF membrane was blocked with 3% bovine serum albumin (BSA) in Tris-Buffered Saline Tween-20 (TBST) Buffer containing 20 mM Tris-HCl,150 mM NaCl and 0.05% Tween 20 overnight at 4°C. The membrane was washed three times with TBST and incubated for 1 h at 37°C with rabbit anti-DEV polyclonal antibody and rabbit pre-serum at 1:100 of dilution in TBST buffer containing 0.5% BSA. After washing, the membrane was incubated for 1 h at 37°C with anti-rabbit-HRPO conjugated IgG(GE Healthcare Limited, Buckinghamshire, United Kingdom) at 1:5000 of dilution in TBST buffer containing 0.5% BSA. Finally, the membrane was washed with TBST and placed in diamino benzidine (DAB) solution as a chromogen to visualize the binding.

### Indirect ELISA

Flat bottomed 96 well plate (Corning, Corning Costar Corp., MA, USA) was coated overnight at 4°C with the recombinant proteins at 7 ug/ml in carbonate bicarbonate buffer, pH 9.6. After blocking with 1% BSA in PBS, the wells were washed with PBS containing 0.05% (v/v) Tween-20 (PBST) and later incubated at 37°C for 1 h with various dilutions (1:5120-1:10) of duck anti-DEV strain Chv positive serum and negative serum. After washing with PBST, the plates were incubated at 37°C for 1 h with anti-duck-HRPO conjugated IgG(GE Healthcare Limited, Buckinghamshire, United Kingdom). Finally, the plate was washed and the peroxidase reaction was visualized by using tetrame-thylbenzidine(TMB) (Sigma) as substrate after incubation for 10 min at room temperature. The reaction was stopped by adding 2 M H_2_SO_4 _and absorbance was read at 450 nm by a microplate autoreader (Bio-Rad).

### Production of polyclonal antibody

Preimmune serum was collected prior to immunization. New Zealand white rabbits were immunized firstly intradermally with a mixture of 1 mg purified recombinant rVP19c or rVP19c(N) mixed with an equal volume of complete Freund's adjuvant (Sigma). Two weeks later, the rabbits were boosted twice subcutaneously with the same amount of recombinant proteins mixed with an equal volume of incomplete Freund's adjuvant at a one-week interval. Two weeks after the last immunization, the two antiserums were harvested from the carotid artery. Then the polyclonal antibody was purified by protein A affinity IgG purification kit according the user's guide. The titer of the specific antibodies was determined by ELISA.

### Indirect immunofluorescent assay

For immunofluorescence assays, monolayers of duck embryo fibroblast (DEF) cells were infected (MOI 5) with the DEV strain Chv and then incubated for 30 h at 37°C. Cells were fixed with 4% paraformaldehyde for 30 min, washed with PBS and permeabilized with 0.5% Triton X-100 for 10 min. After washing, the cells were blocked with PBS containing 5% BSA for 1.5 h at 37°C. Subsequently, the cells were incubated with rVP19c-specific polyclonal antibody diluted in PBS containing 0.5% BSA for 1 h at 37°C. Finally, fluorescein isothiocyanate (FITC)-conjugated goat anti-rabbit immunoglobulin G (Sigma) was added at a dilution of 1:200 and incubated at 4°C for 16 h. After each incubation step, the cells were washed extensively with PBS. The cell nuclei were visualized by DAPI counterstaining [19-21]. The images captured with fluorescence microscopy (Nikon, Japan).

## Competing interests

The authors declare that they have no competing interests.

## Authors' contributions

JX and SCZ carried out most of the experiments and drafted the manuscript. ACC and MSW have critically revised the manuscript and the experimental design. HC, CJS, DKZ, RYJ, QHL, ZLC and XYC helped in experiments. All authors have read and approved the final manuscript.
